# The association between socioeconomic status, psychopathological symptom burden in mothers, and early childhood caries of their children

**DOI:** 10.1371/journal.pone.0224509

**Published:** 2019-10-28

**Authors:** Uta Knoblauch, Gerhard Ritschel, Kerstin Weidner, Sabine Mogwitz, Christian Hannig, Gabriele Viergutz, Maria Lenk

**Affiliations:** 1 Department of Psychotherapy and Psychosomatic Medicine, University Hospital Carl Gustav Carus, Technische Universität Dresden, Dresden, Germany; 2 Policlinic of Dental Maintenance, University Hospital Carl Gustav Carus, Technische Universität Dresden, Dresden, Germany; Medical University of Vienna, AUSTRIA

## Abstract

**Objectives:**

Various maternal mental disorders and socioeconomic status [SES] are discussed as risk factors for early childhood caries [ECC]. In our study, we examined a wide range of symptoms of mental disorders with the aim to identify those maternal psychopathological symptom burdens [PSBs] which show relevant associations with ECC. Our second objective was to investigate how SES affects the associations between PSB and ECC.

**Methods:**

In this study, sixty children with ECC (caries group [CG]) and sixty caries-free children [NON-CG] with their mothers were recruited at two sites in Germany. Children aged three or four years were included in the study. Children’s dental status [dmf-t] and plaque index were recorded, and mothers answered a multidimensional SES index (including education, profession and income) as well as screening questionnaires capturing dental anxiety, depressive disorders, generalized anxiety, somatic symptom burden, eating disorders, traumatic childhood experiences, nicotine dependency and alcohol dependency.

**Results:**

Mothers of the CG reported significantly higher dental anxiety (d_Cohen_ = 0.66), childhood trauma (d_Cohen_ = 0.53) and nicotine dependency (d_Cohen_ = 0.64) than the NON-CG. However, mediator analyses showed that these effects were partly mediated by the SES. Mothers of the CG had a significantly lower SES (d_Cohen_ = 0.93); with education as strongest predictor of dental status. The groups did not differ significantly in symptoms of depressiveness, subjective somatic symptom burden, alcohol dependency, eating disorders, and generalized anxiety.

**Conclusions:**

Several PSBs are associated with ECC, however the SES as the strongest influencing factor mediates this association. Difficult socioeconomic conditions might predispose for both, ECC and mental illness. Targeted strategies are needed to facilitate the use of preventive measures and dental health services especially in families of lower status. For this purpose, psychosocial risk constellations must be identified. More integrative, multifactorial oriented research is necessary to gain a bio-psycho-social understanding of ECC.

## Introduction

Early childhood caries (ECC) is one of the most common chronic diseases in childhood [1]. It is defined as the occurrence of at least one carious lesion on a deciduous tooth within the first 71 months of life [2] and can lead to complete destruction of the primary dentition and impairment of the permanent teeth [3]. In Germany, the prevalence of ECC has decreased in broad parts of the population [4]. According to the DAJ-Study, a recent representative national survey in Germany, 13.7% of the 3-year-old children already had caries experience, while by average, 3.57 teeth were affected per child and only 26% of the carious teeth were restored sufficiently [4]. A polarization of the disease is observed to the extent that a small part of the population accounts for the main caries load. This part of the population could not be reached sufficiently by prevention strategies [4,5]. As a reason for the high caries prevalence in this group, a constellation of low SES and suboptimal oral hygiene and preventive behavior is suspected [4]. A multifactorial disease development model is assumed for ECC [6]. Increasing importance is attributed to psychosocial risk factors such as maternal psychopathologies [7–24], educational deficits and poverty [15]. However, the still fragmentary knowledge about the influence of psychopathologies and their widely neglected interactions with socioeconomic factors complicates the integration in a multifactorial bio-psycho-social model. In most previous studies only one or few disorders were considered, making it difficult to compare the effects of the influencing factors. A few of these determinants are quite well verified (e.g., parental smoking [17–20,25], dental anxiety [7–10,21–24]), others have hardly (e.g., alcohol [11]) or not at all been investigated (e.g., childhood trauma, eating disorders), or study results are contradictory (e.g., depressiveness: positive studies [10,12–14], negative studies [15,16]).

Among the maternal psychopathologies, dental anxiety is one of the most studied risk factors. In children of mothers suffering from dental anxiety, an increased caries prevalence was confirmed in the majority of studies [7–10,21–24]. Mothers’ dental fear can impede the dental care of their children in two ways: apart from avoiding own dental visits, dentally anxious mothers take their children later and less regularly to the dentist [9]. Moreover, maternal dental anxiety predisposes for the development of dental fear in children [24,26].

Although maternal dental fear is a risk factor of ECC [7–10,21–24], nothing is known in this context about the influence of childhood traumatization, which is a common cause of dental anxiety in females [27]. Particularly traumatized women, who experienced sexual abuse in their childhood, often suffer from dental anxiety even in adulthood and perceive dental visits as intimidating [28,29]. A German study on inpatients with mental diseases showed that 42% of patients with posttraumatic stress disorder due to abuse and neglect in childhood suffered from high dental anxiety [30]. Women with childhood traumatization report inadequate dental care in their own childhood [31], more often have dental anxiety [27,28] and have issues with mother-infant bonding [32]. This leads to the assumption that maternal traumatization has a potential yet overlooked impact on ECC.

To better understand the causal relationships between maternal psychopathological symptom burden [PSB] and ECC, the socioeconomic framework conditions of the families should also be considered. Studies have shown that both, mental disorders [33,34] as well as ECC [5,35–41], are associated with low SES, which implicates SES could be a linking factor. Such a mediating influence of the SES is suspected [19] as an explanation for the observed association between household smoking and ECC [17–20,25]. However, since studies focusing on the influence of PSB on ECC often record the SES insufficiently or not at all, little is known about the triangular relationships of these factors. Such methodical limitations in the assessment of the SES are, for instance, the use of unvalidated questionnaires, very few or even only single items yielding little information.

Overall, previous research has confirmed that many maternal psychosocial burdens are associated with children’s oral health. On this basis, the next important step for further research would be to verify the relevance of these influencing factors and to understand the interactions between psychopathological and socioeconomic aspects. The integration into a multifactorial bio-psycho-social model seems necessary to identify risk constellations for need-adapted prevention strategies.

Therefore, the specific objective of our study was to cover a broad spectrum of maternal psychopathologies in order to identify those that show significant associations with ECC and to clarify if these associations are either direct or confounding effects, mediated by the common influence of SES on ECC and PSB. The aim of our study was to examine the following hypotheses:

1. Mothers of children with ECC have a higher symptom burden in various mental disorders than mothers of caries-free children
2. Mothers of children with ECC differ from mothers of caries-free children regarding SES.
3. The SES affects the association between PSB and ECC.

## Methods

### Study population and sampling procedure

Sixty children with ECC (caries group [CG]) and sixty caries-free children [NON-CG] with their respective mothers were recruited at two sites in Germany (University Hospital Dresden and a dental practice in Görlitz, each n = 30 per group). Children aged three or four years were included in the study. The NON-CG had no carious teeth (decayed-missing-filled-teeth [dmf-t] index = 0]) while children with at least 4 untreated carious teeth met the criteria for the CG (dmf-t ≥ 4). Physically and/or mentally disabled as well as chronically ill children, mothers with insufficient understanding of the German language and mothers younger than 18 years were excluded from the study. During routine examinations the children’s dental status and plaque-index were assessed. For ethical reasons, we performed no examinations that only served research purposes. Mothers received a set of self-report questionnaires to collect data on their PSB, SES, oral hygiene habits and dental health care utilization of mother and child. The clinical examination took place at two sites in Germany and was performed by two dentists. Dentist 1 examined the children at the Clinic for Restorative Dentistry at the University Hospital Dresden, while Dentist 2 examined all children at a dental practice in Görlitz. In order to avoid systematic deviations by the locations or examiners, the same number of children was recruited for CG and NON-CG per site (30 children per group at each site). To ensure a high concordance between the examiners, the dental examinations of the first 20 children were carried out in the presence of both dentists.

The study was conducted in accordance with the guidelines of the World Medical Association Declaration of Helsinki (Version 2008) and was approved by the ethics committee at the Technische Universität Dresden (EK 289082014). Research was performed with the understanding and written consent of all legal guardians, normally the parents.

### Clinical examination

The dental examinations were performed using dental mirrors and probes after drying the teeth with compressed air. Each tooth (t) was assessed according to the dmf-t index as either decayed (d), missing (m) or filled (f). If a tooth met one of these criteria, it was assigned a value of 1 whereby the total dmf-t sum score could range between 0 and 20 [42]. Children with dmf-t = 0 were included in the NON-CG and children with dmf-t ≥ 4 were assigned to the CG.

Additionally, a plaque index [PI] was recorded to evaluate the effectivity and regularity of domestic oral hygiene [43]. A modified version of the Greene and Vermillion Oral Hygiene Index (Debris Index) [44] adapted to the deciduous dentition, the behavior and endurance of pre-school children was used. The plaque extension was evaluated in thirds of the dental crown: 0: no plaque, 1: plaque on the gingival third of the clinical crown, 2: plaque on the gingival and on parts of the medium third of the clinical crown, or 3: plaque on more than two thirds of the clinical crown. The measurements per tooth were summed up (range: 0 to 60) and divided by the number of existing teeth (total range: 0 to 3).

### Questionnaire

PSB was assessed with different validated and internationally established questionnaires. Mothers were asked about their socioeconomic status (socioeconomic status index [SES index]) [45], dental anxiety (Dental Anxiety Scale [DAS]) [46], depressive disorders (Patient Health Questionnaire-8 [PHQ-8]) [47], generalized anxiety (Generalized-Anxiety-Disorder-7 [GAD-7]) [48], somatic symptom burden (Somatic-Symptom-Scale-8 [SSS-8]) [49], eating disorders (SCOFF) [50], traumatic childhood experiences (Childhood Trauma Screener [CTS]) [51], nicotine dependency (Fagerström Test for Nicotine Dependence [FTND]) [52], and alcohol abuse (Alcohol Use Disorders Identification Test [AUDIT]) [53]. Furthermore, data on domestic oral hygiene habits and dental examinations was obtained from several self-designed items (for detailed information about the study procedure, the instruments and additional references see supplementary material: S1 File).

### Statistical analysis

Prior to the study, the required minimum number of cases was estimated. We aimed to identify clinically relevant influencing factors. As relevant effects, at least medium effect sizes were considered. 50 persons per group are recommended as the optimum sample size in order to ensure the detection of medium effects (d = 0.5) when comparing two equally large, independent groups with a significance level of 5% and a power (1-β) of 0.8 [54]. Since individual missing values have to be taken into account when using questionnaire scales, we recruited n = 60 mother-child pairs per group.

Statistical analyses were carried out using SPSS 24 (IBM Corp., Armonk, NY). An imputation of missing data was not performed. Potential variations from the total N are reported. Non-parametric group comparisons were used because some of the variables did not show normal distributions.

Group differences of ordinal and metric variables were examined using Mann-Whitney-U tests and of categorical variables using Fisher’s exact tests. Spearman correlations were used as measures of association. Descriptive values were stated as median [$\tilde{x}$] and quartiles [*x*_.25_; *x*_.75_]. As measure of effect size, we used Cohen’s d.

First, CG and NON-CG were compared for clinical parameters, SES and all PSB questionnaires using U-tests. For all those symptoms both groups significantly differed in (DAS, FTND, CTS) two mediation models were performed using PROCESS v3 by Andrew Hayes [55]. The dependent variables (Y) were dmf-t (model 1) and plaque-index PI (model 2). The respective psychopathology served as independent variable (X), and SES as the mediation variable (M). Total, indirect and direct effects were reported.

## Results

### Sample characteristics

There are no significant differences between both groups concerning **maternal age** (U-test: $z=1.331;\;p=0.183;\text{NON}-\text{CG}:\tilde{x}=\;32.0\;years,\;x_{.25}\;=\;30.0\;years,\;x_{.75}\;=\;35.0\;years;\text{CG}:\;\tilde{\text{x}}=\;34.0\;years,\;x_{.25}\;=\;30.0\;years,\;x_{.75}\;=\;38.0\;years$), **marital status** (Fisher-exact: *p* = 0.287) and **partnership status** (Fisher-exact: *p* = 0.735). Significant group-differences were found regarding **nationality**, with a higher proportion of non-German mothers in the CG (Fisher-exact: *p* = 0.017; German: NON-CG 98,3% (*n* = 59), CG 85.0% (*n* = 51); other nationality: NON-CG 1.7%(*n* = 1), CG 15.0%(*n* = 9)). In both groups, in median two children under the age of 14 years were living per household (U-test: *z* = 0.694; *p* = 0.488).

### Clinical parameters

**Fig 1:**
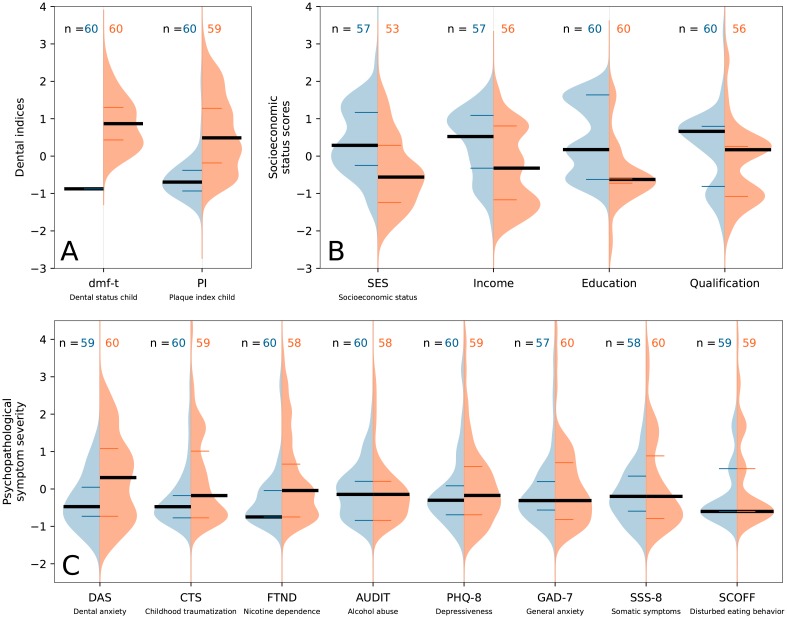
Psychopathological symptom burden, SES and dental parameters in the CG and NON-CG. For each of the (z-transformed) variables, the probability distribution (kernel density estimation) is depicted for the mothers of the NON-CG (left, blue) and the mothers of the CG (right, red). Additionally, the medians (bold black lines) as well as the 25^th^ and the 75^th^ percentiles (narrow colored lines) of the distributions are shown. **A**: dmf-t and plaque index of the children in both groups. **B**: Total SES and its subscales of the mothers of both groups. **C**: Psychopathological symptom severities of the mothers of both groups, measured with different screening questionnaires.

The median dmf-t was 8 in the CG (range [4;16]; *x*_.25_ = 6; *x*_.75_ = 10). No tooth ($\tilde{x}$) was missing (range [0;5]), no tooth was filled (range [0;5]) and 6 teeth (range [4;14]) were decayed, which results in a restoration-rate of $\tilde{x}=0\%$ (*x*_.75_ = 0%; range [0.0%; 56.0%] in the CG. Furthermore, the plaque-index PI was significantly increased in the CG compared to the NON-CG (Table 1, Fig 1A).

**Table 1:** Group characteristics and comparison for dental parameters, SES and PSB.

Scale	NON-CG percentiles			CG percentiles			U-test			Effect size	Correlation with status		Correlation with education	
	25.	50.	75.	25.	50.	75.	n	z	p	*d*_*Cohen*_	*r*_*S*_	p	*r*_*S*_	p
**Dental parameters**														
dmf-t	0	0	0	6.00	8.00	10.00	120				-0.442	<0.001	-0.492	<0.001
PI	0.10	0.25	0.45	0.55	1.00	1.50	119	7.20	<0.001	1.57	-0.444	<0.001	-0.521	<0.001
**Socioeconomic status (SES)**														
Status	11.15	13.10	16.25	7.70	10.10	13.20	110	4.47	<0.001	0.93				
Education	3.60	4.80	7.00	3.15	3.60	3.68	120	5.47	<0.001	1.06				
Profession	2.40	4.05	4.20	2.10	3.50	3.60	116	3.25	<0.001	0.48				
Income	3.50	5.00	6.0	2.00	3.50	5.50	113	4.07	<0.001	0.80				
**Psychopathologies**														
DAS	7.00	8.00	10.00	7.00	11.00	14.00	119	2.99	0.003	0.66	-0.321	0.001	-0.306	0.001
GAD-7	2.00	3.00	5.50	1.00	3.00	7.00	117	0.51	0.606	0.14	0.038	0.696	0.029	0.757
PHQ-8	2.00	3.50	5.00	2.00	4.00	7.00	119	1.09	0.313	0.18	-0.041	0.675	0.015	0.867
CTS	1.00	1.20	1.40	1.00	1.40	2.20	119	2.25	0.025	0.53	-0.167	0.080	-0.283	0.002
SCOFF	0.00	0.00	1.00	0.00	0.00	1.00	118	1.48	0.139	0.36	-0.145	0.135	-0.208	0.024
SSS-8	3.00	5.00	8.00	2.00	5.00	11.50	118	0.35	0.725	0.27	-0.118	0.135	-0.081	0.383
AUDIT	0.00	2.00	3.00	0.00	2.00	3.00	118	0.87	0.385	0.12	-0.048	0.619	-0.012	0.897
FTND*	1.00	1.00	2.00	1.00	2.00	3.25	118	3.21	0.001	0.64	-0.393	<0.001	-0.448	<0.001

Note: dental status (dmf-t), plaque index (PI), dental anxiety (DAS), generalized anxiety (GAD-7), depressive disorders (PHQ-8), traumatic childhood experiences (CTS), eating disorders (SCOFF), somatic symptom burden (SSS-8), alcohol abuse (AUDIT), nicotine dependency (FTND* Non-smokers included). For both groups (NON-CG and CG) and all scales percentiles, group differences (U-test), effect sizes (d_Cohen_) and associations with social status and education (Spearman rank correlation) are presented.

### Domestic oral hygiene habits and utilization of dental care

The results of the mothers’ self-reports about oral hygiene and utilization of dental care are shown in Table 2. Based on the mothers’ information, children’s teeth in the NON-CG were brushed significantly more often per day compared to the CG. However, mothers of both groups did not differ significantly regarding their own tooth-brushing frequency, but tooth-brushing frequencies of mother and child were correlated significantly. Almost all children in both groups used toothpaste and received additional help from adults when brushing their teeth. At their first dental visit, children of the CG were significantly older than children in the NON-CG $\left(n=114;z=2.048;\;p=0.041;Non-CG:\;\tilde{x}=1\;year;\;x_{.25}=1\;year;\;x_{.75}=2\;years;CG:\;\tilde{x}=2\;years;\;x_{.25}=1\;year;\;x_{.75}=3\;years\right)$ and there were significant differences concerning the reason for the first consultation. The NON-CG reported more often that their child visited the dentist to get familiar with the dental office. The CG named more frequently pain or other symptoms as the reason for the first visit. There was no significant difference between the CG and the NON-CG concerning the proportion of children who visited the dentist at least once a year for a checkup. But significantly more mothers from the CG replied that they themselves visit a dentist only irregularly or only in case of problems. A significant but weak correlation between the maternal and the child’s frequency of dental visits could be shown (compare Table 2).

**Table 2:** Mothers’ self-reports about their own and their children’s oral hygiene and utilization of dental services.

Question	NON-CG		CG		p	Correlation behavior mother—child	
	%	n	%	n		r_s_	p
**Toothbrushing: frequency child**					0.031	0.305	0.001
Not regularly	1.7	1	5.0	3			
Once per day	3.3	2	15.0	9			
Twice per day or more often	95.0	57	80.0	48			
**Toothbrushing: frequency mother**					0.101		
Not regularly	0.0	0	5.0	3			
Once per day	5.1	3	10.0	6			
Twice per day or more often	94.9	56	85.0	51			
**Child uses toothpaste**					1.000		
Yes	100.0	60	98.3	59			
No	0.0	0	1.7	1			
**Toothbrushing is performed by**					0.573		
Child	5.0	3	1.7	1			
Child with the help of adults	91.7	55	91.7	55			
Adults	3.3	2	6.7	4			
**Reason for first dental visit**					<0.001		
Familiarization with dental practice	54.2	32	33.3	20			
Consultation/Prophylaxis	44.1	26	36.7	22			
Pain/other symptoms	1.7	1	26.7	16			
Referral	0.0	0	3.3	2			
**Frequency of dental visits: child**					0.114	0.215	0.019
≥ once per year for check-up	98.3	57	90.0	54			
Unregularly (in case of problems)	1.7	1	10.0	6			
**Frequency of dental visits: mother**					0.002		
≥ once per year for check-up	96.7	58	76.7	46			
Unregularly (in case of problems)	3.3	2	23.3	14			

Note: Absolute frequencies (n) and percentages per group as well as results of the group comparison using Fisher’s exact test (p values) are reported.

### Maternal SES and PSB

Mothers in the CG had a significantly lower SES than the NON-CG (see Table 1 and Fig 1B). Group differences were significant in all 3 SES domains. In both groups, about one third of all mothers were the main earner of their household and there were no significant differences between both groups regarding the mode of employment. Descriptive information about the socioeconomic conditions (SES level, mode of employment, main earner of the household) and results of the group comparisons are shown in Table 3.

**Table 3:** Socioeconomic conditions in both groups (p-value for group comparison with Fisher exact test).

	CG		NON-CG		p	n
	%	n	%	n		
**SES level**					<0.001	110
Low	26.4	14	3.5	2		
Medium	54.7	29	52.6	30		
High	18.9	10	43.9	25		
**Mode of employment**					0.061	120
Full-time	28.3	17	26.7	16		
Part-time	36.7	22	55.0	33		
Occasional/irregular jobs	10.0	6	10.0	6		
unemployed	25.0	15	8.3	5		
**Main earner: mother**	35.0	21	26.7	16	0.429	120

Group comparisons (U-tests) of the maternal PSB showed no significant differences concerning depressiveness (PHQ-8), generalized anxiety (GAD-7), subjective somatic symptom burden (SSS-8), eating disorders (SCOFF) and alcohol dependency (AUDIT). However, mothers of the CG had significantly higher rates of childhood traumatization (CTS), nicotine dependency (FTND) and dental anxiety (DAS) than mothers of the NON-CG (see Table 1 and Fig 1C). 21.7% (*n* = 13) of the CG and 3.4% (*n* = 2) of the NON-CG suffered from high dental anxiety (*DAS* ≥ 15). For DAS and CTS, a low but significant correlation was shown (*n* = 118; *r*_*S*_ = 0.220; *p* < 0.017).

Dental parameters (dmf-t, PI) as well as those maternal PSBs in which the groups differed were significantly correlated with SES or at least with the subscale education (Spearman correlation coefficients are shown in Table 1). These triangular relations were further examined with mediation analyses. Mediation models confirmed the significant influence of maternal PSB in the areas dental anxiety (DAS), childhood traumatization (CTS) and nicotine dependence (FTND) on the child’s dmf-t (≙ total effect). However, these effects were partially mediated by the SES (indirect effect). The share of the total beta was between 1/3 and 1/2 in each case. None of the three maternal PSBs had a significant influence on the child’s plaque-index (total and direct effects p>0.05, see Table 4).

**Table 4:** Results of the mediation models.

Model	Total effect				Direct effect				Indirect effect			R^2^
	B	95% CI		p	B	95% CI		p	B	95% CI		
**X: dental anxiety [DAS]**	0.308	0.131	0.486	0.001	0.198	0.023	0.373	0.027	0.110	0.040	0.198	0.219
**Y: dental status [dmf-t]**												
**M: socioeconomic status [SES]**												
**X: childhood traumatization [CTS]**	0.223	0.046	0.400	0.014	0.145	-0.021	0.312	0.086	0.078	0.007	0.160	0.208
**Y: dental status [dmf-t]**												
**M: socioeconomic status [SES]**												
**X: nicotine abuse [FTND]**	0.319	0.138	0.500	0.001	0.191	0.010	0.373	0.039	0.128	0.059	0.215	0.221
**Y: dental status [dmf-t]**												
**M: socioeconomic status [SES]**												
**X: dental anxiety [DAS]**	0.130	-0.063	0.324	0.185	0.013	-0.178	0.204	0.892	0.117	0.044	0.204	0.141
**Y: plaque index [PI]**												
**M: socioeconomic status [SES]**												
**X: childhood traumatization [CTS]**	-0.005	-0.193	0.183	0.956	-0.084	-0.262	0.094	0.352	0.079	0.005	0.158	0.151
**Y: plaque index [PI]**												
**M: socioeconomic status [SES]**												
**X: nicotine abuse [FTND]**	0.186	-0.011	0.383	0.063	0.053	-0.145	0.251	0.596	0.133	0.061	0.233	0.151
**Y: plaque index [PI]**												
**M: socioeconomic status [SES]**												

Psychopathologies served as independent variable (X), SES as the mediation variable (M), and dental status or plaque index as dependent variable (Y). Total, indirect and direct effects are reported.

## Discussion

### Association between maternal PSB and ECC

A multifactorial disease development model is assumed for ECC [6]. Increasing importance is attributed to psychosocial risk factors, such as maternal psychopathologies [7–24], educational deficits and poverty [15]. However, the still fragmentary knowledge about these influencing factors complicates the development of a multifactorial bio-psycho-social understanding of the disease. In contrast to previous studies, we did not focus on the influence of an individual maternal PSB; instead, we included a wide range of potential psychosocial risk factors to identify those having a relevant impact on ECC. As clinically relevant effects we considered at least medium effect sizes (d = 0.5), on which our sample size estimation was based (n = 120). Utilizing validated and in international research well-established screening questionnaires allowed us to assess maternal symptom burden of different psychopathologies. Out of all factors, three PSBs, in which mothers of the CG scored significantly higher than mothers of the NON-CG, could be identified. Group differences with medium effect sizes were observed for dental anxiety (DAS: d_Cohen_ = 0.66), childhood traumatization (CTS: d_Cohen_ = 0.53) and nicotine dependency (FTND: d = 0.64). An influence of maternal dental anxiety and smoking on ECC has already been proven in several studies. Previous research confirmed the association between smoking in the household and ECC [17–20] as well as an increased caries prevalence in children of dentally anxious mothers [7–10,21–24]. In our study, 22% of the mothers in the CG, compared with 3% in the NON-CG, suffered from high dental anxiety. In contrast, the association between maternal childhood traumatization and ECC has been unknown, although it seems plausible that experienced abuse or neglect can impede mothers to take adequate care of their children’s oral health. It is known that women with childhood traumatization report inadequate dental care in their own childhood [31], often suffer from dental anxiety, perceive dental visits as intimidating [28,29] and more often have issues with mother-infant bonding [32]. Both childhood traumatization and dental anxiety are associated with avoidance behavior [27,56]. Mothers’ avoidance of dental treatment carries the risk that their children will also have less access to preventive measures [57]. In our CG, fewer mothers made use of regular dental checkups and mother’s and child’s frequency of dental visits showed significant associations.

We also examined PSBs, that were previously investigated rarely (e.g., alcohol [11]), not at all (e.g., eating disorders, subjective impairments due to somatic symptoms), or with contradictory evidence (e.g., depressiveness: positive studies [10,12–14], negative studies [15,16]). Our results confirm no significant group differences between CG and NON-CG with respect to alcohol dependency, symptoms of eating disorders, general anxiety, depressiveness or somatic symptom burden. However, our findings do not exclude that small effects may exist, which were not detected due to the sample size of our study. In two studies with higher power a relationship between depressiveness and ECC was found [12,14].

### SES mediates the relationship between PSB and ECC

After identifying relevant maternal PSBs, we aimed to understand their interactions with socioeconomic factors. In our study the SES had the strongest effect out of all influencing factors (d_Cohen_ = 0.93). Furthermore, the results of the mediation analyses demonstrated that the effects (beta weights) of the PSBs are considerably reduced when including SES as a mediator, which means that confounding effects play a role. Thus, mental disorders seem to be associated with ECC less directly than previously assumed. Difficult socioeconomic conditions might predispose for both ECC and mental illness. It is well known that PSB [33,34] and ECC [35–41,58] are related with SES. Within the SES domains, mothers’ educational level had the strongest effect on ECC (d_Cohen_ = 1.06). A similar conclusion was drawn by Finlayson et al., who found a caries-preventive effect of education and a general overrepresentation of maternal depressiveness (35%) in a cohort of low-income families, while depressiveness was not directly associated with ECC [15]. In a similar way, a mediating influence of SES is presumed [19] for the relationship between smoking in the household and ECC, which has already been observed in several studies [17–20,25]. However, although the significance of the risk factor SES is known and a mediating function between PSB and ECC is suspected, this interaction has hardly been investigated. As one reason for the fact that SES, contrary to its importance, is often treated as a marginal topic, we suspect the uncertainties and methodological problems that can be noticed in the assessment of socioeconomic factors (such as use of unvalidated questionnaires, very few or even only single items yielding little information). In order to address the complex construct of SES better, multidimensional instruments were developed like the index used in our study. Such indices capture several status domains (usually: education, qualification and income) in a more detailed fashion. Today, they are preferred in large health surveys [59], because they allow to quantify the influence of the separate domains and usually also provide a metric total score for the SES, which makes several subsequent analyses (ANCOVA, mediation/moderation analyses) possible [45]. The use of validated instruments also enables comparability between studies and comparison with standard values of representative samples.

### Limitations and outlook

In conclusion, our study indicates the need for a more multifactorial bio-psycho-social perspective in further research. Our results show that some maternal PSBs are significantly associated with ECC and that the SES as the main risk indicator mediates this association. A better understanding of this triangular relationship is necessary to identify risk constellations for need-adapted prevention strategies. However, we are limited by the power of our study. Further research is necessary to examine the interactions between relevant influencing factors and to identify risk constellations and potential confounders (e.g., using structural equation models, directed acyclic graphs or cluster analyses). This requires valid instruments for assessing the SES, which are comparable and usable even in complex statistical models.

Some other limitations of our study should be noted. Our cross-sectional design permits no causal interpretations. Due to the regional recruitment our study is no representative survey. Furthermore, the transferability of the results to other nations should be examined in further research. The recruitment in dental practices may have caused a selection bias, as we could not reach families who avoid dental treatments. The voluntary nature of participation may also have an impact. The participants were examined at two different locations and by two dentists. However, as this was conducted in equal parts in both groups, group comparisons should not be affected by systematic errors. Socially desirable response behavior cannot be ruled out, especially regarding the responses on domestic oral hygiene. For assessing childhood traumatization retrospectively by self-report, a memory bias cannot be excluded [60].

Although we cannot conclude any causal interpretations, low socioeconomic status seems to forward the emergence of ECC. It is of importance that preventive programs also reach socially deprived, lower educated families in order to improve the domestic oral hygiene and preventive health care utilization. For this purpose, also the identification and a professional handling of maternal dental anxiety and traumatization is important, because it can restrain them from regular dental visits or receiving instructions on adequate oral hygiene for themselves and their children. Dentists are often consulted by these families too late. However, doctors of other disciplines (e.g., pediatricians, psychiatrists, gynecologists) and psychotherapists, who are treating mothers and their infants, could contribute to the prevention and early detection of ECC. For this reason, and especially in high-risk groups, health practitioners should ask their patients for oral problems and dental health care utilization.

## Supporting information

S1 File Supplementary material. Additional information about the instruments used in the study and sensitivity analysis.
(DOCX)

S2 File Data set. (XLSX)
