# Supplementary material for: The association between socioeconomic status, psychopathological symptom burden in mothers, and early childhood caries of their children
Source: PLoS One. 2019 Oct 28;14(10):e0224509. doi: 10.1371/journal.pone.0224509 (PMC6816547; doi:10.1371/journal.pone.0224509)
Supplement: S1 File — Additional information about the instruments used in the study and sensitivity analysis. (DOCX) [file pone.0224509.s001.docx]

**Supplementary material**

**1. Additional information about the instruments used in the study**

**Questionnaires:**

Socioeconomic status [SES]:

The socioeconomic-status index [1] is a modified version of the German Cardiovascular Prevention Study’s (GCP) [2] social-screening index [3], first used in the 2009 GEDA study. The SES is a multidimensional index that assesses the subscales “*education”*, “*profession”* and “*income”*. Each subscale is captured using multiple items. The response categories are assigned scores, so that each subscale takes a quasi-metric value in the range of 1.0 to 7.0 points. The sum of the three subscales form the so-called SES Index, which can take values between 3.0 and 21.0 points [1].

The subscale *“education”* consists of two items capturing schooling and vocational training of the participant. For the subscale *“profession”*, the professional statuses of the participant and of the principal earner (of the household) are captured. The subscale *“income”* yields a so-called net-equivalent income, which is determined by the net-household income and the need-weights of all household inhabitants [1].

The total SES index values are categorized in the following way:

low SES: 3.0 to 7.9,

medium SES: 8.0 to 13.8,

high SES: 13.9 to 21.0 [1].

Data on domestic oral hygiene habits and dental examinations:

Mothers (participating with their child in our study) were asked about the domestic implementation of dental preventive measures (toothbrushing frequency and -assistance) and the history of dental visits (reasons for former visits and regularity of checkups) of mother and child.

Dental Anxiety Scale [SES]:

The DAS is a validated screening instrument for dental anxiety [4]. Patients are supposed to imagine four common dental treatment situations and rate their perceptions on a five-point scale. Scores between 4 and 20 points can be reached. In our study, the German version [5] was used.

Patient Health Questionnaire [PHQ-8]:

The PHQ-8 [6] assesses depressive symptoms and is a revised version of the PHQ-9 [7] without a suicide/self-injury item. The PHQ-9 items capture the diagnose criteria of the Major Depression in DSM-IV. Total scores range between 0 and 24 points.

Generalized Anxiety Disorder-7 [GAD-7]:

The GAD-7 assesses symptoms of generalized anxiety disorder according to DSM-IV [8]. The seven items are answered on a four-point Likert scale and deal with typical symptoms of generalized anxiety the participant experienced during the last two weeks. Total scores range between 0 and 21 points. An internal consistency (Cronbach’s α) of 0.92 and a test-retest-reliability of 0.83 were reported [9].

Somatic Symptom Scale-8 [SSS-8]:

The SSS-8 is a self-assessment questionnaire with eight questions. It was developed as a short version of the PHQ-15 [10] for the DSM-V [11]. On a five-point Likert scale, ranging from 0 (“not at all”) to 4 (“very strong”), the participant chooses the statement that describes his/her symptoms during the last 7 days. A good internal consistency is reported (α = 0.81) [12].

SCOFF:

The acronym “SCOFF” stands for the first letters of the terms sick, control, one, fat and food, which are used in the original version of the questionnaire. SCOFF is a screening instrument for bulimia nervosa and anorexia nervosa and consists of 5 dichotomous questions. If two or more items are answered with “yes”, the subject is likely to suffer from one of these disorders [13].

Childhood Trauma Screener [CTS]:

The CTS [14] is a short questionnaire derived from the subscales of the Childhood Trauma Questionnaire (CTQ) [15]. It is used to identify traumatic experiences in the participant’s childhood and youth. The internal consistency is high (α = 0.724). All questions are answered on a five-point Likert-scale.

Fagerström Test for Nicotine Dependence [FTND]:

The FTND was developed from the Fagerström Tolerance Questionnaire [16,17]. The FTND assesses relevant criteria for nicotine addiction and is used to determine the severity of nicotine dependence. A total score of 0 to 10 points can be reached and five severity categories are defined. The internal consistency is α = 0.61 [18].

In our study non-smokers and former smokers were included in the variable (we named the variable FTND*). The encoding of the variable is shown in table 1.

Table 1: Encoding of the variable for nicotine dependence in our study [FTND*]

| **Category** | **Code** |
| --- | --- |
| Non-smoker | 1 |
| Former smoker | 2 |
| None / very low nicotine dependency (FTND 0-2) | 3 |
| Low nicotine dependency (FTND 3-4) | 4 |
| Medium nicotine dependency (FTND 5) | 5 |
| Strong nicotine dependency (FTND 6-7) | 6 |
| Very strong nicotine dependency (FTND 8-10) | 7 |

Alcohol Use Disorders Identification Test [AUDIT]:

The AUDIT is used to detect excessive alcohol consumption and -abuse [19]. With 10 items the questionnaire assesses drinking behavior, alcohol consumption and alcohol-related problems. The total score ranges between 0 and 40 points. For women, a total score of 7 or more indicates dangerous or harmful alcohol consumption [20].

**Oral parameters:**

Clinical examination:

Clinical examinations were performed at the department for pediatric dentistry of the University Hospital C. G. Carus Dresden and in one of its co-operative practices in Görlitz. The children were seated on the examination chair alone or with their parents. Dental records of children treated under general anesthesia were reevaluated on the day of the dental restoration. Missing and filled teeth were recorded, followed by the visual assessment of dental plaque and carious lesions. No radiographs were used for caries detection.

dmf-t index:

The dmf-t index sums up the number of deciduous teeth with caries-experience [21] and stands for decayed/missing/filled deciduous teeth. If a tooth meets one of these criteria, it is assigned a value of 1. Total scores between 0 and 20 can be recorded in the primary dentition.

Plaque-index:

Plaque-indices are used to quantify the biofilm adhering to the teeth’s smooth surfaces and to evaluate the effectivity and regularity of domestic oral hygiene [22]. In our study, a modified version of the Greene and Vermillion Oral Hygiene Index (Debris Index) [23] adapted to the deciduous dentition, the behavior and endurance of pre-school children was used. The buccal surfaces of all teeth were dried with compressed air and checked for visible plaque by using a dental mirror and dental probe. The plaque extension was evaluated in thirds of the dental crown: 0: no plaque, 1: plaque on the gingival third of the clinical crown, 2: plaque on the gingival and on parts of the medium third of the clinical crown, or 3: plaque on more than two thirds of the clinical crown. The measurements per tooth were summed up (range: 0 to 60) and divided by the number of existing teeth (total range: 0 to 3).

For ethical reasons, no additional examinations, which would have served for research purposes only, or which are not recommended for 3- to 4-year-old children [24], were performed (e.g., use of plaque-revelators, periodontal probing, time-consuming assessment of oral surfaces).

**2. Sensitivity analysis**

A post-hoc sensitivity analysis for two-sided Mann-Whitney-U tests with two groups, both of size N = 60, shows a minimum detectable effect size of (Cohen's) d = 0.53 for our study (under the assumptions α = 0.05 and β = 0.2). This means that our group comparisons should be able to detect medium to large effects.

**References**

1. Lampert T, Kroll LE, Müters S, Stolzenberg H. Measurement of the socioeconomic status within the German Health Update 2009 (GEDA). Bundesgesundheitsblatt - Gesundheitsforsch - Gesundheitsschutz. 2013;56: 131–143. doi:10.1007/s00103-012-1583-3

2. Troschke J v., Herz-Kreislauf-Präventionsstudie FD. Die deutsche Herz-Kreislauf-Präventionsstudie: Design und Ergebnisse. Bern-Göttingen-Toronto-Seattle: Huber; 1998.

3. Winkler J. Die Messung des sozialen Status mit Hilfe eines Index in den Gesundheitssurveys der DHP. In: Ahrens W, Bellach B, Jöckel K (Hrsg. ., editors. Messung soziodemographischer Merkmale in der Epidemiologie RKI Schriften 1/98. München: MMV Medizin Verlag; 1998. pp. 69–74.

4. Corah NL. Development of a dental anxiety scale. J Dent Res. 1969;48: 596. doi:10.1177/00220345690480041801

5. Margraf-Stiksrud J, Sergl HG. Angst und Angstabbau. In: Sergl HG, editor. Psychologie und Psychosomatik in der Zahnheilkunde. München: Urban und Schwarzenberg; 1996. p. 297.

6. Kroenke K, Strine TW, Spitzer RL, Williams JBW, Berry JT, Mokdad AH. The PHQ-8 as a measure of current depression in the general population. J Affect Disord. 2009;114: 163–73. doi:10.1016/j.jad.2008.06.026

7. Kroenke K, Spitzer RL, Williams JB. The PHQ-9: validity of a brief depression severity measure. J Gen Intern Med. 2001;16: 606–13.

8. Saß H, Houben I. Diagnostisches und statistisches Manual psychischer Störungen. DSM-IV. Göttingen: Hogrefe Verlag GmbH & Co. KG; 1996.

9. Spitzer RL, Kroenke K, Williams JBW, Löwe B. A brief measure for assessing generalized anxiety disorder: the GAD-7. Arch Intern Med. 2006;166: 1092–7. doi:10.1001/archinte.166.10.1092

10. Kroenke K, Spitzer RL, Williams JBW. The PHQ-15: validity of a new measure for evaluating the severity of somatic symptoms. Psychosom Med. 2002;64: 258–266.

11. Falkai P, Wittchen H-U. Diagnostisches und statistisches Manual psychischer Störungen DSM-5. 1. Auflage. Göttingen: Hogrefe Verlag GmbH & Co. KG; 2015.

12. Gierk B, Kohlmann S, Kroenke K, Spangenberg L, Zenger M, Brähler E, et al. The somatic symptom scale-8 (SSS-8): a brief measure of somatic symptom burden. JAMA Intern Med. 2014;174: 399–407. doi:10.1001/jamainternmed.2013.12179

13. Morgan JF, Reid F, Lacey JH. The SCOFF questionnaire: a new screening tool for eating disorders. West J Med. 2000;172: 164–165. doi:10.1136/ewjm.172.3.164

14. Grabe HJ, Schulz A, Schmidt CO, Appel K, Driessen M, Wingenfeld K, et al. [A brief instrument for the assessment of childhood abuse and neglect: the childhood trauma screener (CTS)]. Psychiatr Prax. 2012;39: 109–15. doi:10.1055/s-0031-1298984

15. Bernstein DP, Fink L, Handelsman L, Foote J, Lovejoy M, Wenzel K, et al. Initial reliability and validity of a new retrospective measure of child abuse and neglect. Am J Psychiatry. 1994;151: 1132–6. doi:10.1176/ajp.151.8.1132

16. Heatherton TF, Kozlowski LT, Frecker RC, Fagerström KO. The Fagerström Test for Nicotine Dependence: a revision of the Fagerström Tolerance Questionnaire. Br J Addict. 1991;86: 1119–27.

17. Fagerstrom KO, Schneider NG. Measuring nicotine dependence: a review of the Fagerstrom Tolerance Questionnaire. J Behav Med. 1989;12: 159–82.

18. Bleich S, Havemann-Reinecke U, Kornhuber J. FTNA - Fagerström-Test für Nikotinabhängigkeit. 1. Auflage. Göttingen: Beltz Test GmbH; 2002.

19. Saunders JB, Aasland OG, Babor TF, de la Fuente JR, Grant M. Development of the Alcohol Use Disorders Identification Test (AUDIT): WHO Collaborative Project on Early Detection of Persons with Harmful Alcohol Consumption--II. Addiction. 1993;88: 791–804.

20. Institut Suchtprävention. AUDIT ( Alcohol Use Disorders Identification Test ): Selbsttest Alkohol [Internet]. Linz; 2016 [cited 1 Jan 2018] p. 40. Available: https://www.praevention.at/sucht-vorbeugung/suchtmittel/alkohol/alkohol-selbsttest.html

21. Gängler P, Hoffmann T, Willershausen B, Schwenzer N, Ehrenfeld M. Konservierende Zahnheilkunde und Parodontologie: 66 Tabellen. 3. unverän. Stuttgart: Thieme; 2010.

22. Kramer E. Grundlagen zur Zahngesundheit; mit 21 Tabellen. 10. überar. Köln: Deutscher Zahnärzte Verlag; 2009.

23. Greene JC, Vermillion JR. The oral hygiene index: a method for classifying oral hygiene status. J Am Dent Assoc. 1960;61: 172–177.

24. Hellwege K-D. Die Praxis der zahnmedizinischen Prophylaxe: ein Leitfaden für die Individualprophylaxe, Gruppenprophylaxe und initiale Parodontaltherapie. 6th ed. Stuttgart; New York: Georg Thieme Verlag; 2003.
